# Association of cognitive impairment and elderly mortality: differences between two cohorts ascertained 6-years apart in China

**DOI:** 10.1186/s12877-020-1424-4

**Published:** 2020-01-28

**Authors:** Jun Duan, Yue-Bin Lv, Xiang Gao, Jin-Hui Zhou, Virginia Byers Kraus, Yi Zeng, Hong Su, Xiao-Ming Shi

**Affiliations:** 1 Department of Epidemiology and Health Statistics, School of Public Health, Anhui Medical University, 81 Meishan Road, Hefei, 230032 Anhui Province China; 2 National Institute of Environmental Health, Chinese Center for Disease Control and Prevention, #7 Panjiayuan Nanli, Chaoyang, Beijing, 100021 China; 3 Nutritional Epidemiology Lab, Pennsylvania State University, Philadelphia, PA USA; 4 Duke Molecular Physiology Institute and Division of Rheumatology, Department of Medicine, Duke University School of Medicine, Durham, NC USA; 5 Center for the study of Aging and Human Development and the Geriatric Division of School of Medicine, Duke University, Durham, NC USA; 6 Center for Study of Healthy Aging and Development Studies, Peking University, Beijing, China

**Keywords:** Aging, Cohort study, Cognitive impairment, Oldest old, Mortality

## Abstract

**Background:**

Cognitive impairment is a major contributor to mortality among the elderly. However, the relationship between cognitive impairment evaluated by educational levels and mortality and the trend between cognitive impairment and mortality with time are unclear. We aim to evaluate the differences in associations of cognitive impairment, taking the stratification by educational levels into account, with all-cause mortality and further explore the relationship of cognitive impairment with mortality in different age and sex groups in two cohorts ascertained 6 years apart in China.

**Methods:**

A total of 13,906 and 13,873 Chinese elderly aged 65 years and older were included in the 2002–2008 and 2008–2014 cohorts from the Chinese Longitudinal Healthy Longevity Survey (CLHLS). Mortality data was ascertained from interviews with family members or relatives of participants. Cognitive function, evaluated by the Mini-Mental State Examination (MMSE), were defined by different cut-offs taking educational background into account. Cox models were used to explore the relationship of cognitive impairment with mortality.

**Results:**

For the 2002–2008 and 2008–2014 cohorts, 55,277 and 53,267 person-years were followed up, and the mean (SD) age were 86.5 (11.6) and 87.2 (11.3) years, respectively. Compared to normal cognition, cognitive impairment was independently associated with higher mortality risk after controlling for potential confounders, with hazard ratios (HRs) of 1.32 (95% confidence interval [CI], 1.25–1.39) in 2002–2008 cohort and 1.26 (95% CI, 1.19–1.32) in 2008–2014 cohort, stratified by educational levels. The trend of cognitive impairment with all-cause mortality risk decreased from 2002 to 2008 to 2008–2014 cohort, while no significant interaction of cognitive impairment with cohort for all-cause mortality was observed. The associations of cognitive impairment and mortality were decreased with age in the two cohorts.

**Conclusions:**

Cognitive impairment evaluated by different cut-offs were associated with increased risk of mortality, especially among those aged 65–79 years in the two cohorts; this advocates that periodic screening for cognitive impairment among the elderly is warranted.

## Introduction

Cognitive impairment is a major risk factor for poor health in the growing population of elders worldwide [1–3]. It imposes a heavy burden on public health and is associated with shortened life expectancy. The prevalence of mild cognitive impairment aged 65 years or older in China was about 20.8% in 2014 [4]; more than half of these individuals progresses to dementia within 5 years [5]. As China, the world’s largest developing country quickly transitions into an aging society, it was reported that the mortality attributable to dementia in China increased from 1.6 million in 1990 to 2.3 million in 2016 [6], which can profoundly impact Chinese elderly health-related quality of life and longevity.

Although a number of epidemiological studies have reported on a cognitive impairment-mortality relationship [3, 7], most of them concerned the association of cognitive impairment and mortality risk of elders in high-income contries [8–12]. In upper-middle income countries such as China, several studies have indicated that baseline cognitive impairment increases the risk of all-cause mortality [13, 14]. Earlier studies have found that education is strongly related with cognitive performance [15]. In China, illiteracy is still widely prevalent, particularly among the elderly population [16]. In previous studies, the prevalence of cognitive impairment was different for entire cohort according to the cut-offs of education, which is higher than reported previously in China [16]. Therefore, it’s necessary to consider cognitive impairment, stratified by different cut-offs taking educational background into account when we investigate the relationship between cognitive impairment and mortality in the elderly population. In addition, women have a higher risk of cognitive impairment, while a lower risk of death may lead to gender differences. The relationship between cognitive impairment and mortality risk have been extensively reported and the results were inconsistent [13, 17]. Moreover, studies are limited that include large sample sizes, national representation, different age groups in the oldest old (aged 80 and older).

Prior studies have explored the association of cognitive impairment and all-cause mortality risk with long-term follow-up (14 years and 20 years) [13, 17], but have rarely studied the impacts of change in medical, demographic and social factors over time on the association between cognitive impairment and mortality. It has been reported that annual mortality among the oldest old was substantially declined between 0.2 and 1.3% in 1998–2008 compared with the participants of the same age born before 10 years, but cognitive impairment increased annually between 0.7 and 2.2% in the past ten years [18]. Therefore, it is unclear whether the impact of cognitive impairment on mortality has changed with the passage of time.

The present study aims to examine and compare the relationship between cognitive impairment, stratified by different cut-offs taking educational background into account, and mortality using two cohort studies conducted in 2002–2008 and 2008–2014. Moreover, subgroup analyses were further conducted among different sex and age groups to identify susceptible populations in 2002–2008 and 2008–2014.

## Materials and methods

### Study design and participants

The Chinese Longitudinal Healthy Longevity Survey (CLHLS) was a nationwide survey that randomly selected half of the cities and counties in 23 provinces of China, and recruited participants aged 65 years and older. A more detailed description of the CLHLS has been published elsewhere [19].

The protection of human subjects for the CLHLS was approved by the Ethics Committees. All participants or their legal representatives signed written consent forms to participate in the baseline and follow-up surveys.

### Assessment of cognitive impairment and mortality

The present study evaluated baseline cognitive status of two cohorts, ascertained in 2002 and 2008, using the same scale of cognitive function. Cognitive impairment was evaluated using the Mini-Mental State Examination (MMSE), a widely used cognitive test [20] and adapted into the Chinese language based on the international standard of MMSE questionnaire, and carefully tested by previous pilot survey interviews [21]. The total MMSE score ranges from 0 to 30 within 6 dimensions: orientation, registration, attention, language, memory, and visual construction skills. Three methods were used to define cognitive impairment: (1) ≥24, 18–23, and < 18 were used to define normal cognition (reference), mild cognitive impairment and serious cognitive impairment [22, 23]; (2) < 18 was used to define cognitive impairment for participants who didn’t receive any formal education, < 21 for participants who received 6 years of education or less, and < 25 for participants who received more than 6 years of education [16, 24]; (3)≥24 and < 23 were used to define normal cognition (reference) and cognitive impairment [22, 23].

The main outcome was all-cause mortality occurring during the follow-up survey in 2002–2008 and 2008–2014, with followed up every 3 years respectively. Each cohort was followed for 6 years to quantify mortality and the mortality date. Mortality date was ascertained from interviews with family members or relatives of participants [18]. The cause-specific mortality was not involved in this study because (1) many of the elderly died at home rather than in medical institutions where cause of mortality might be recorded, and (2) mortality surveillance systems are uncertain in many survey fields.

### Assessment of potential confounding variables

A number of variables were collected through a face-to-face standardized questionnaire, including demographic characteristics, economic status, lifestyles, health conditions and medical services.

Marital status was classified into unmarried and married. Education level was classified as no formal education, elementary school graduate (1–6 years of education), and high school graduate (>6 years of education). Region was defined as: urban, rural and suburban. Exercise was categorized into yes or no. Housework and reading were divided into 3 categories: never, sometimes, and often. Binary variables were defined to assess current status of smoking, drinking, depression and disability in six activities of daily living (ADL) including dressing, bathing, using the toilet, getting in/out of a bed or chair and feeding. An ADL impairment was defined as a elder’ s response of “needs help” to at least one or more of activities associated with one of the six items. Participants with systolic blood pressure ≥ 140 mmHg or diastolic blood pressure ≥ 90 mmHg were considered hypertensive. Self-reported history of heart disease and stroke were also collected. According to the survey, we assessed the level of medical care by whether the participant was receiving adequate medical care at present? (Yes or No)” and on the basis of the payor of the medical costs (public medicare or not)”. We assessed economic status by asking, “are all financial sources enough for your life?”, “do you have a pension?” and “how is your life compared with other local people? (richer, similar and poorer)”.

### Statistical analysis

We divided the elderly into 3 groups by the conventional MMSE cut-off points for 2002–2008 and 2008–2014. Mean and standard deviation were summarized for continuous variables, frequency and percentage for categorical variables. Comparisons between the elderly were conducted using the chi-square test for categorical variables, Kruskal-Wallis test for continuous variables.

Kaplan–Meier analysis was used to draw the survival curves stratified by MMSE score, compared by the log-rank test. We used the Cox proportional hazards models and the important confounders were identified by previous studies [13, 17]. Less than 1.3% for all independent variables had missing values, and due to such low missing rates, we deleted the cases with missing values, and the results have no significant difference compared to those with imputation [18].

Hazards ratio (HR) and 95% confidence intervals (CIs) were estimated with the construction of Cox proportional hazards models: the crude model was model 1; age and sex were adjusted in model 2; marital status, living alone, exercise, alcohol consumption, and smoking status were further adjusted in model 3; ADL and depression were further adjusted in model 4; and medical care and economic status were further adjusted in model 5. We pooled data from the 2002 cohort and the 2008 cohort and a variable that was assigned a value of 0 in the 2002 cohort and 1 in the 2008 cohort was included in the Cox proportional hazards model [25]. We tested the interaction between each cohort and cognitive impairment and explored whether the influence of cognitive impairment on mortality decreases over time. For subgroup analyses, the elderly were stratified by age group (65–79, 80–89, 90–99, and ≥ 100) and sex (male and female) in model 5. Interactions of baseline cognitive impairment with age group and sex on mortality risk were explored.

We cannot measure long-term progression about cognitive function on mortality. Previous studies demonstrated that both cognitive impairment and disability were independent of adverse impact on mortality [26, 27]. Therefore, the interaction synergistic effects between ADLs and cognitive impairment were tested in the models.

The following sensitivity analyses were conducted to check the robustness of the primary results: (1) removed the participants lost to follow-up to examine possible attrition bias; (2) excluded survival time less than 1 year due to the possibility that disease status in the last year of life could have affected the risk effects; (3) additionally adjusting for place of residence, dietary habits, hypertension, self-reported heart disease and stroke.

Data analysis was conducted using R version 3.3.1. All statistical tests were 2-sided, and statistical significance was judged by *P*-values <0.05.

## Results

### Baseline characteristics

**Fig. 1:**
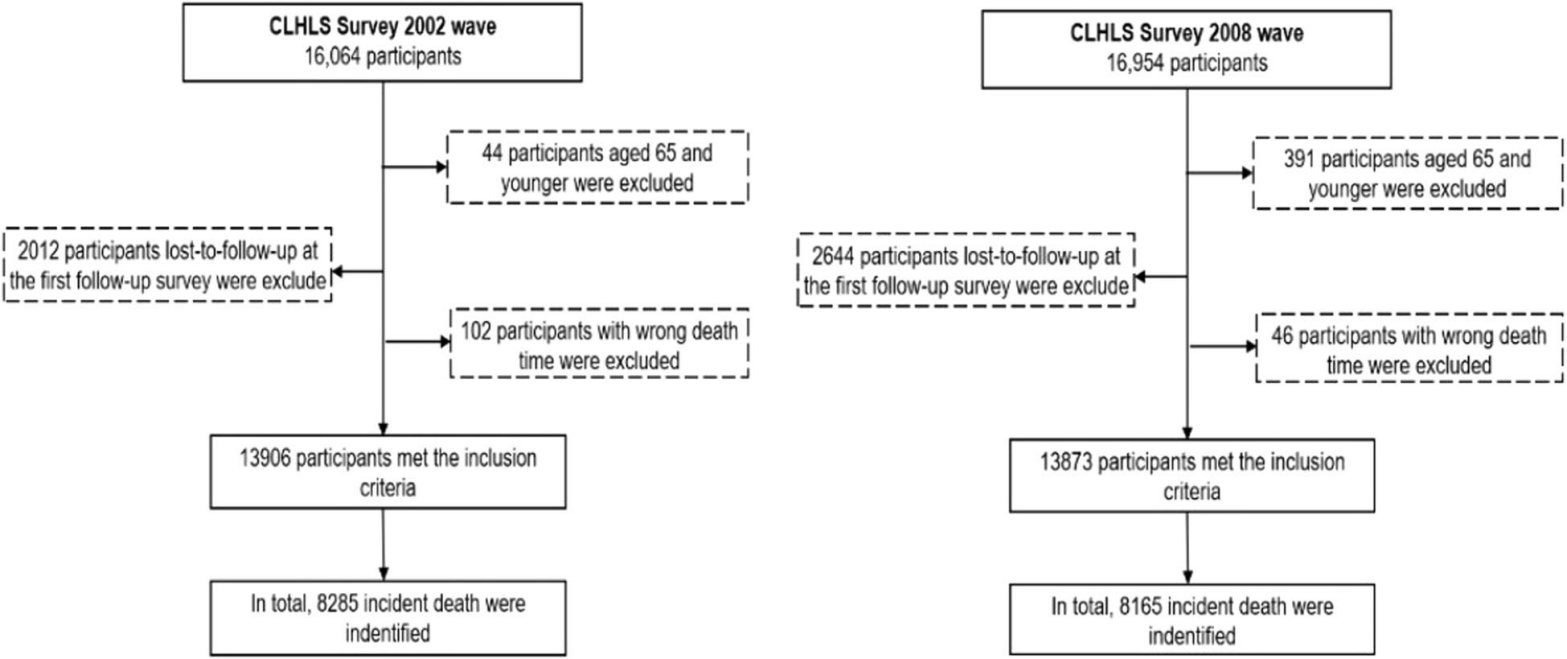
Flowchart of the study population, the Chinese Longitudinal Healthy Longevity Survey (CLHLS) form 2002–2008 (Left) and 2008–2014 (Right)

A total of 13,906 and 13,873 elderly aged 65 and older participated in the baseline survey in 2002 and 2008. The mean (SD) age of the elderly were 86.52 (11.63) and 87.22 (11.33) years in the two cohorts. A total of 55,277 person-years were documented in the 2002–2008 cohort, and 53,267 in the 2008–2014 cohort. Characteristics of the elderly who survived, died or lost to follow-up are displayed in Fig. 1. Participants with cognitive impairment tended to be older; female; unmarried; less likely to smoke, drink and live alone; have lower baseline ADL and depression; have higher rates of inadequate medical care and no pension. The baseline conditions of the two cohorts were similar (Table 1).

**Table 1:** Baseline characteristics of the study participants according to MMSE scores

Characteristic	2002–2008					2008–2014				
	MMSE ≥ 24	18 ≤ MMSE<24	0<MMSE<18	N(%)	*P* value	MMSE ≥ 24	18 ≤ MMSE<24	0<MMSE<18	N(%)	*P* value
Age	81.60 (10.7)	90.11 (9.8)	95.76 (7.9)	86.52 (11.6)	<.001	82.17 (10.6)	89.75 (9.1)	95.44 (7.9)	87.22 (11.3)	<.001
Survival status					<.001					<.001
Died	3630 (44.7)	1685 (71.0)	2970 (87.2)	8285 (59.6)		3360 (43.3)	1282 (66.2)	3523 (84.5)	8165 (58.9)	
Censored	4497 (55.3)	689 (29.0)	435 (12.7)	5621 (40.4)		4409 (56.8)	654 (33.8)	645 (15.5)	5708 (41.1)	
Sex					<.001					<.001
Male	4196 (51.6)	796 (33.5)	937 (27.5)	5929 (42.6)		4096 (52.7)	696 (36.0)	1112 (26.7)	5904 (42.6)	
Female	3931 (48.4)	1578 (66.5)	2468 (72.5)	7977 (57.3)		3673 (47.3)	1240 (64.1)	3056 (73.3)	7969 (57.4)	
Education level					<.001					<.001
No formal education	1569 (19.3)	175 (7.4)	204 (6.0)	1948 (14.0)		1723 (22.2)	145 (7.5)	213 (5.1)	2081 (15.0)	
1–6 years	2336 (28.7)	475 (20.0)	488 (14.3)	3299 (23.7)		2116 (27.2)	357 (18.4)	496 (11.9)	2969 (21.4)	
Above 7 years	4222 (52.0)	1724 (72.6)	2713 (79.7)	8659 (62.3)		3930 (50.6)	1434 (74.1)	3459 (83.0)	8823 (63.6)	
In marriage	4616 (56.8)	1893 (79.7)	3064 (90.0)	9573 (68.8)	<.001	4422 (56.9)	1499 (77.4)	3695 (88.7)	9616 (69.3)	<.001
Living alone	1350 (16.6)	471 (19.8)	609 (17.9)	2430 (17.5)	<.001	515 (6.6)	333 (17.2)	2404 (57.7)	3252 (23.4)	<.001
Current smoking	11,323 (22.1)	402 (16.9)	382 (11.2)	2583 (18.6)	<.001	1740 (22.4)	270 (14.0)	455 (10.9)	2465 (17.8)	<.001
Current drinking	1976 (24.0)	454 (19.0)	499 (15.0)	2929 (21.0)	<.001	1622 (21.0)	307 (16.0)	531 (13.0)	2460 (18.0)	<.001
Exercise	4821 (59.3)	1809 (76.2)	2904 (85.3)	9534 (68.6)	<.001	5015 (64.6)	1488 (76.9)	3611 (86.6)	10,114 (72.9)	<.001
ADL impaired	6899 (84.9)	1517 (63.9)	1338 (39.3)	9754 (70.1)	<.001	7224 (93.0)	1545 (79.8)	2193 (52.6)	10,962 (79.0)	<.001
Depression	1941 (23.9)	878 (37.0)	923 (27.1)	3742 (26.9)	<.001	2125 (27.4)	749 (38.7)	1064 (25.5)	3938 (28.4)	<.001
Adequate medical	1916 (7.0)	232 (10.2)	214 (6.2)	2362 (17.4)	<.001	1744 (22.3)	163 (8.2)	298 (7.4)	2205 (16.3)	<.001
Having pension	2030 (25.2)	256 (11.3)	251 (7.1)	2537 (18.4)	<.001	1676 (22.4)	153 (8.5)	276 (7.3)	2105 (15.2)	<.001
Adequate funds	7479 (92.1)	2064 (87.3)	2749 (81.2)	12,292 (88.1)	<.001	7290 (94.4)	1766 (91.3)	3759 (90.1)	12,815 (92.3)	<.001
Self-reported economic status					<.001					<.001
Rich	1604 (20.3)	366 (15.3)	409 (12.2)	2379 (17.3)		1151 (15.3)	233 (12.1)	441 (11.4)	1825 (13.1)	
Common	6361 (78.0)	1907 (80.2)	2808 (80.3)	11,076 (80.0)		6419 (82.7)	1634 (84.2)	3500 (83.6)	11,553 (83.2)	
Poor	162 (1.7)	101 (4.5)	188 (7.5)	451 (2.7)		199 (2.0)	69 (3.7)	227 (5.0)	495 (3.7)	

Note: Values are *n* (%) unless otherwise noted; *MMSE* Mini-Mental State Examination, *ADL* activities of daily living

*P* value for 2002–2008 and 2008–2014, respectively

### Relationship between cognitive impairment and all-cause mortality

Additional file 1: Figure S1 shows the Kaplan-Meier survival curve by different categories of MMSE-based cognitive impairment. Significant differences were revealed by the log-rank test in the 3 groups (cognitive normal, mild cognitive impairment and serious cognitive impairment; *P* < 0.001) among the two cohorts. Table 2 shows that risk of mortality increased in parallel with a decrease in MMSE scores. In the crude model, the elderly with cognitive impairment (MMSE < 18) had an elevated risk of all-cause mortality compared to the other MMSE categories in 2002–2008 (MMSE < 18 [crude HR, 3.56; 95% CI, 3.39–3.74]) and 2008–2014 ([crude HR, 3.25; 95% CI, 3.10–3.41]). After adjusting for sex and age (model 2), demographic characteristics (model 3), functional status (model 4), and medical care and economic status (model 5), similar associations were found between cognitive impairment and mortality in the two cohorts. In the fully adjusted model, participants with MMSE scores indicative of cognitive impairment had increased all-cause mortality risk compared with the participants with normal cognition: for 2002–2008 mild cognitive impairment HR 1.28 (95% CI 1.20–1.37), severe cognitive impairment HR 1.48 (95% CI 1.39–1.57); for 2008–2014 mild cognitive impairment HR 1.20 (95% CI 1.12–1.28), severe cognitive impairment HR 1.32 (95% CI 1.25–1.41) (Table 2). When cognitive impairment was stratified by educational levels, cognitive impairment was independently associated with higher mortality risk, with hazard ratios (HRs) of 1.32 (95% confidence interval [CI], 1.25–1.39) in 2002–2008 cohort and 1.26 (95% CI, 1.19–1.32) in 2008–2014 cohort, compared to normal cognition. Similar results were obtained when cognitive impairment was defined by MMSE<24 or considering education level in both cohorts.

**Table 2:** Hazard ratios (95% CI) for all-cause mortality according to baseline MMSE Score

MMSE Score	Death	Hazard Ratio(95% CI)				
		Model1	Model2	Model3	Model4	Model5
2002–2008						
Cognitive impairment by Education	5069 (49.9)	1(Reference)	1(Reference)	1(Reference)	1(Reference)	1(Reference)
MMSE by Education	3216 (85.7)	2.88 (2.75,3.01)	1.49 (1.41,1.56)	1.44 (1.37,1.51)	1.33 (1.26,1.40)	1.32 (1.25,1.39)
MMSE 24 to 30	3630 (45.3)	1(Reference)	1(Reference)	1(Reference)	1(Reference)	1(Reference)
MMSE<24	4655 (81.8)	2.85 (2.72,2.97)	1.54 (1.47,1.62)	1.48 (1.41,1.56)	1.39 (1.32,1.46)	1.38 (1.31,1.45)
MMSE 24 to 30	3630 (45.3)	1(Reference)	1(Reference)	1(Reference)	1(Reference)	1(Reference)
MMSE 18 to 23	1685 (71.6)	2.12 (2.00,2.25)	1.38 (1.30,1.47)	1.34 (1.26,1.42)	1.29 (1.21,1.37)	1.28 (1.20,1.37)
MMSE<18	2970 (87.4)	3.56 (3.39,3.74)	1.69 (1.60,1.79)	1.62 (1.53,1.72)	1.48 (1.40,1.58)	1.48 (1.39,1.57)
2008–2014						
Cognitive impairment by Education	4454 (47.1)	1(Reference)	1(Reference)	1(Reference)	1(Reference)	1(Reference)
MMSE by Education	3711 (84.2)	2.80 (2.68,2.92)	1.53 (1.45,1.60)	1.37 (1.30,1.44)	1.29 (1.22,1.36)	1.26 (1.19,1.32)
MMSE 24 to 30	3630 (45.3)	1(Reference)	1(Reference)	1(Reference)	1(Reference)	1(Reference)
MMSE<24	4805 (79.2)	2.73 (2.61,2.86)	1.52 (1.44,1.59)	1.37 (1.30,1.44)	1.30 (1.23,1.37)	1.27 (1.20,1.34)
MMSE 24 to 30	4409 (43.4)	1(Reference)	1(Reference)	1(Reference)	1(Reference)	1(Reference)
MMSE 18 to 23	1282 (66.2)	1.92 (1.80,2.05)	1.30 (1.22,1.39)	1.25 (1.17,1.34)	1.22 (1.14,1.30)	1.20 (1.12,1.28)
MMSE<18	3523 (85.1)	3.25 (3.10,3.41)	1.65 (1.56,1.74)	1.46 (1.38,1.55)	1.36 (1.28,1.45)	1.32 (1.25,1.41)

Note: The values of cognitive impairment are n and mortality rate in brackets(%); *CI* confidence interval, *MMSE* Mini-Mental State Examination, *ADL* activities of daily living. Cognitive impairment by Education: < 18 was used to define cognitive impairment for participants who didn’t receive any formal education, < 21 for participants who received 6 years of education or less, and < 25 for participants who received more than 6 years of education

*Hazard ratio (95% CI) was calculated from Cox models

Model1: Unadjusted

Model2: Age + sex

Model3: Model2 + Marry+Living alone+Educational level + Exercise+Smoke+Drink

Model4: Model3 + ADL + Depression

Model5: Model4 + Medical service + Economic status

The association of cognitive impairment with all-cause mortality over the time was decreased comparing the 2002–2008 cohort to the 2008–2014 cohort. However, no significant interaction of cognitive impairment for all-cause mortality among two cohorts was significant (Table 3, *P* > 0.05 for interaction).

**Table 3:** The subgroup analyses of hazard ratios (95% CI) for all-cause mortality according to baseline MMSE score

	MMSE	2002–2008	*P*-interaction	2008–2014	*P*-interaction
Cohort			*/*		*P* = 0.254
	MMSE 18 to 23	1.28 (1.20,1.37)		1.20 (1.12,1.28)	
	MMSE<18	1.48 (1.39,1.57)		1.32 (1.25,1.41)	
Sex			*P* = 0.663		*P* = 0.810
Male	MMSE 18 to 23	1.26 (1.14,1.39)		1.20 (1.08,1.34)	
	MMSE<18	1.42 (1.29,1.57)		1.23 (1.11,1.36)	
Female	MMSE 18 to 23	1.31 (1.21,1.42)		1.21 (1.10,1.32)	
	MMSE<18	1.51 (1.40,1.63)		1.40 (1.29,1.51)	
Age			*P* < .001		*P* < .001
65–79	MMSE 18 to 23	1.41 (1.16,1.72)		1.63 (1.29,2.06)	
	MMSE<18	2.15 (1.64,2.83)		1.79 (1.33,2.41)	
80–89	MMSE 18 to 23	1.27 (1.14,1.42)		1.17 (1.04,1.33)	
	MMSE<18	1.75 (1.56,1.97)		1.33 (1.17,1.52)	
90–99	MMSE 18 to 23	1.26 (1.14,1.40)		1.16 (1.05,1.29)	
	MMSE<18	1.44 (1.31,1.59)		1.39 (1.27,1.53)	
100+	MMSE 18 to 23	1.09 (0.97,1.24)		0.94 (0.81,1.09)	
	MMSE<18	1.29 (1.16,1.43)		1.11 (0.99,1.24)	

Note: *CI* confidence interval, *MMSE* Mini-Mental State Examination

Reference: MMSE 24 to 30

*Hazard ratio (95% CI) was calculated from cox models after adjust age, sex, marry, living alone, educational level, exercise, smoke, drink, ADL, depression, medical service and economic status

### Subgroup analysis

Lower MMSE score was consistently associated with elevated risk of mortality both in 2002–2008 and 2008–2014. The analysis stratified by sex revealed that cognitive impairment was associated with increased risk of all-cause mortality in females compared to males in 2002–2008 with HR 1.35 (95% CI 1.24–1.46) and in 2008–2014 HR 1.20 (95% CI 1.10–1.32). However, an interaction of cognitive impairment with sex for all-cause mortality was not observed (Table 3, *P* > 0.05 for interaction).

Compared to those with normal cognition, MSSE scores > 24, the younger elderly (65–79 years old) with cognitive impairment had higher risk of all-cause mortality, respectively in the two cohorts (Table 3).

Both cognitive impairment and ADL impairment have been considered important risk factors associated with elderly mortality. During the aging process, cognitive impairment and ADL impairment often co-exist and closely interact (Additional file 1: Table S1, S2).

### Sensitivity analysis

Among two cohorts, there was almost no change in the association between cognitive impairment and all-cause mortality after excluding participants lost to follow-up or with survival time less than 1 year. The association was still robust after further adjustment for potential confounders (Additional file 1: Table S3, S4).

## Discussion

In this large-scale prospective analysis we explored the association of cognitive impairment and all-cause mortality based on two Chinese cohorts with 6 years each of follow-up. Firstly, cognitive impairment evaluated by MMSE score was closely related with an increased risk of all-cause mortality and the risk effect of cognitive impairment on mortality was lower by the cut-off of education level. Secondly, the association of cognitive impairment with risk of all-cause mortality was lower in 2008–2014 versus 2002–2008, but there were no significant interaction of cognitive impairment for all-cause mortality among two cohorts between them. Thirdly, the association of cognitive impairment with all-cause mortality was reduced with age in both cohorts.

A relationship between cognitive impairment and elevated risk of mortality in elders has been reported consistently by epidemiological studies. The MMSE as a general measure of cognitive impairment has been an important predictor of all-cause mortality [28, 29]. To date, although few studies have explored the relationship between cognitive impairment and all-cause mortality in China [30–32], the existing results support our conclusion that cognitive impairment is independently associated with an increased risk of death in the elderly [13, 14]. Moreover, MMSE scores are known to be influenced by education [16]. The risk of cognitive impairment evaluated by MMSE score, stratified by education was lower than used by the same cutoff point as in Western countries. The effect might be overestimated due to the illiterate or less educated in China. Thus, in the near future, as the level of education increases, it is possible that we will be able to re-evaluate by a certain cut-off.

This study discovered that the association of cognitive impairment with the risk value of all-cause mortality has declined among the Chinese elderly in 2008–2014 compared to 2002–2008 and an interaction of cognitive impairment for all-cause mortality among two cohorts was not significant. China has achieved medical insurance coverage for all people since China’s medical reform in 2008. Governments at all levels are increasingly investing in medical reform, such as medical technology and equipment [33]. These benefits might be expected to play a part in reducing mortality from all causes in the later cohort.

Previous studies on sex differences in the association of cognitive impairment with all-cause mortality have been inconsistent [13, 34]; this might be attributed to regional differences and insufficient sample size for analysis. An et al. indicated that males had a higher risk than females, which might be attributed to an undesirable lifestyle among men, such as smoking and drinking [17]. Conversely, Kirsten found that women perform worse than men with respect to lifelong subnormal cognitive functioning or emotional disorders [22]. However, there are also studies that reported no sex-specific differences [13]. Similarly, we did not find sex differences between baseline cognitive impairment and all-cause mortality. It is necessary for further analyses to explore possible different patterns of mortality among sexes with cognitive impairment.

Many population-based studies have reported that cognitive impairment was strongly associated with subsequent mortality in the elderly [22, 35, 36]. Our study was consistent with previous findings and further demonstrated that the association of cognitive impairment with all-cause mortality was more pronounced among younger elderly in two Chinese cohorts. One possible reason that cognitive impairment of younger elderly poses a greater risk of mortality may be due to their ability to develop cognitive impairment faster compared to the oldest old, thus triggering a greater risk of mortality. The varying association of cognitive impairment and all-cause mortality in different age groups might be due to the survival bias that the oldest old represent hardy survivors who have successfully adapted to cognitive impairment [31]. Another possible explanation is that the oldest old have a higher risk of mortality, a common competing risk for cognitive impairment, thus causing loss to follow up bias and confusing the association [13].

Several strengths are worth mentioning in our findings. We included the representative sample to explore the relationship between cognitive impairment, evaluated by different cut-offs, and mortality among the Chinese elderly population based on two 6-year cohorts. Moreover, our age-specific analyses including 65–79, 80–89, 90–99 and ≥ 100 age groups can help us obtain a more comprehensive understanding of the impact of cognitive impairment on risk of mortality in elders. In addition, our sensitivity analyses suggested that the findings of this study were robust.

Some limitations of this study should be acknowledged. Firstly, cognitive impairment was measured using the MMSE (not based on clinical diagnosis) and we were not being able to distinguish between elders with and without dementia. However, we tried to reduce the impact of long-term progression about cognitive function by tested the interaction cognitive impairment and functional impairment. Secondly, despite the effort of adjusting for a number of confounders such as demographic characteristics, lifestyle factors, heath conditions, health service and economic status, we could not completely eliminate the risk of confounding bias due to unobserved differences in personal characteristics. Thirdly, the association of rapid cognitive decline and mortality might differ in the elderly whose cognitive status did not decline or declined slowly. In our study, we only focused on baseline cognitive impairment and did not assess whether cognitive decline over time was associated with elevated risk of mortality.

## Conclusions

The data from this population-based longitudinal study revealed that cognitive impairment was significantly associated with increased risk of all-cause mortality, and the relationship of cognitive impairment, stratified by educational levels with mortality was lower than previous studies. Thus, prevention and management of cognitive impairment taking the educational levels into account might have substantial benefits for mortality in the health policies or clinical practice. .

## Supplementary information

**Additional file 1: Figure S1.** Kaplan-Meier curve for hazard of death by the baseline MMSE score (Left: 2002-2008, Right: 2008-2014). **Table S1.** The subgroup analyses of hazard ratios (95% CI) for all-cause mortality according to baseline MMSE score. **Table S2.** Association of combined cognition-ADL function with all-cause mortality among elders in the 2002-2008 and 2008-2014. **Table S3.** Sensitivity analyses for the association between cognitive impairment (MMSE<24) and all-cause mortality. **Table S4.** Sensitivity analyses for the association between cognitive impairment ( taking into account the educational background) and all-cause mortality.

## Data Availability

This study was based on the datasets from the Chinese Longitudinal Healthy Longevity Survey (CLHLS) in longevity areas. The CLHLS data can be publicly obtained through the National Archive of Computerized Data on Aging (NACDA). (https://www.icpsr.umich.edu/icpsrweb/NACDA/series/487)

## Abbreviations

ADL: Activities of Daily Living
CIs: Confidence Intervals
CLHLS: Chinese Longitudinal Healthy Longevity Survey
HRs: Hazard Ratios
MMSE: Mini-Mental State Examination
