# Supplementary material for: Association of cognitive impairment and elderly mortality: differences between two cohorts ascertained 6-years apart in China
Source: BMC Geriatr. 2020 Jan 28;20:29. doi: 10.1186/s12877-020-1424-4 (PMC6988297; doi:10.1186/s12877-020-1424-4)
Supplement: Supplementary file 1 — Additional file 1: Figure S1. Kaplan-Meier curve for hazard of death by the baseline MMSE score (Left: 2002-2008, Right: 2008-2014). Table S1. The subgroup analyses of hazard ratios (95% CI) for all-cause mortality according to baseline MMSE score. Table S2. Association of combined cognition-ADL function with all-cause mortality among elders in the 2002-2008 and 2008-2014. Table S3. Sensitivity analyses for the association between cognitive impairment (MMSE<24) and all-cause mortality. Table S4. Sensitivity analyses for the association between cognitive impairment ( taking into account the educational background) and all-cause mortality. [file 12877_2020_1424_MOESM1_ESM.doc]

**
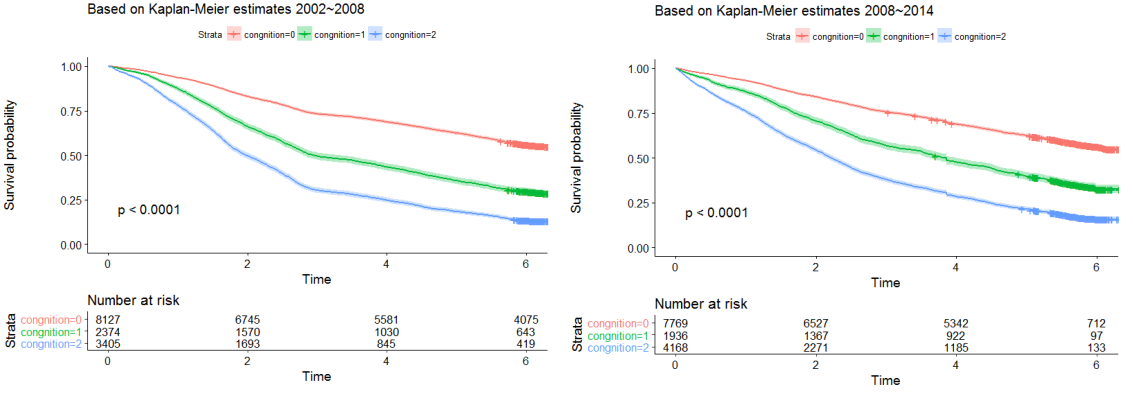
**

**Figure S1.** Kaplan-Meier curve for hazard of death by the baseline MMSE score (Left: 2002-2008，Right: 2008-2014).

**Table S1.** The subgroup analyses of hazard ratios (95% CI) for all-cause mortality according to baseline MMSE score.

| **ADL Status** | **2002-2008** | **2008-2014** |
| --- | --- | --- |
| ADL normal | 1.31(1.22,1.41) | 1.24(1.16,1.32) |
| ADL impaired | 1.33(1.24,1.43) | 1.30(1.18,1.43) |
| P-interaction | *P*<.001 | *P*<.001 |

Note: CI, confidence interval; ADL, activities of daily living.

Reference: Cognitive impairment by Education: <18 was used to define cognitive impairment for participants who didn’t receive any formal education, <21 for participants who received 6 years of education or less, and <25 for participants who received more than 6 years of education.

*Hazard ratio (95% CI) was calculated from Cox models after adjust age, sex, marry, living alone, educational level, exercise, smoke, drink, depression, medical service and Economic status.

**Table S2.** Association of combined cognition-ADL function with all-cause mortality among elders in the 2002-2008 and 2008-2014.

| Groups by the cognitive and physical function | **2002-2008** | *P*-value | **2008-2014** | *P*-value |
| --- | --- | --- | --- | --- |
| neither cognitive nor functional impairment | 1（Reference） |  | 1（Reference） |  |
| no cognitive impairment, but functional impairment | 1.42(1.33,1.52) | <0.01 | 1.31(1.23,1.40) | <0.01 |
| Cognitive impairment but no functional impairment | 1.55(1.45,1.65) | <0.01 | 1.48(1.36,1.61) | <0.01 |
| cognitive and functional impairment | 1.92(1.80,2.05) | <0.01 | 1.83(1.70,1.96) | <0.01 |

Note: CI, confidence interval; ADL, activities of daily living.

*Hazard ratio (95% CI) was calculated from Cox models after adjust age, sex, marry, living alone, educational level, exercise, smoke, drink, depression, medical service and Economic status.

**Table S3.** Sensitivity analyses for the association between cognitive impairment (MMSE＜24) and all-cause mortality.

|  | 2002-2008 | 2008-2014 |
| --- | --- | --- |
| Excluding loss to follow-up | 1.37(1.30,1.44) | 1.27(1.20,1.34) |
| Excluding mortality in the first year | 1.37(1.30,1.45) | 1.26(1.19,1.34) |
| Adjusting for Urban and Rural | 1.38(1.31,1.45) | 1.25(1.19,1.32) |
| Adjusting for Housework | 1.36(1.27,1.41) | 1.26(1.16,1.29) |
| Adjusting for Read | 1.36(1.29,1.43) | 1.25(1.18,1.32) |
| Adjusting for hypertension | 1.38(1.31,1.45) | 1.27(1.20,1.34) |
| Adjusting for heartdisease | 1.38(1.31,1.46) | 1.27(1.25,1.36) |

Note: CI indicates confidence interval; MMSE, Mini-Mental State Examination.

Reference: MMSE 24 to 30.
*Hazard ratio (95% CI) was calculated from Cox models after adjust age, sex, marry, living alone, educational level, exercise, smoke, drink, ADL, depression, medical service and Economic status.

**Table S4.** Sensitivity analyses for the association between cognitive impairment ( taking into account the educational background) and all-cause mortality.

|  | 2002-2008 | 2008-2014 |
| --- | --- | --- |
| Excluding loss to follow-up | 1.30(1.22,1.44) | 1.25(1.18,1.31) |
| Excluding mortality in the first year | 1.31(1.24,1.38) | 1.26(1.19,1.30) |
| Adjusting for Urban and Rural | 1.32(1.25,1.40) | 1.26(1.19,1.32) |
| Adjusting for Housework | 1.32(1.26,1.41) | 1.25(1.16,1.29) |
| Adjusting for Read | 1.31(1.24,1.39) | 1.26(1.18,1.31) |
| Adjusting for hypertension | 1.31(1.25,1.39) | 1.27(1.20,1.32) |
| Adjusting for heartdisease | 1.32(1.25,1.40) | 1.26(1.22,1.31) |

Note: CI indicates confidence interval; MMSE, Mini-Mental State Examination.

Reference: Cognitive impairment by Education: <18 was used to define cognitive impairment for participants who didn’t receive any formal education, <21 for participants who received 6 years of education or less, and <25 for participants who received more than 6 years of education.
*Hazard ratio (95% CI) was calculated from Cox models after adjust age, sex, marry, living alone, educational level, exercise, smoke, drink, ADL, depression, medical service and Economic status.
